# Breast Cancer Risk Factors among Women with Solid Breast Lesions

**DOI:** 10.3390/clinpract14020036

**Published:** 2024-03-18

**Authors:** Ivana Eremici, Andreea Borlea, Catalin Dumitru, Dana Stoian

**Affiliations:** 1 PhD School, Victor Babes University of Medicine and Pharmacy, 300041 Timisoara, Romania; 2 Department of Internal Medicine II, Victor Babes University of Medicine and Pharmacy, 300041 Timisoara, Romania; 3 Obstetrics and Gynecology Department, Victor Babes University of Medicine and Pharmacy, 300041 Timisoara, Romania; dumitru.catalin@umft.ro

**Keywords:** breast cancer screening, risk factors, solid breast lesions, women

## Abstract

Background: Breast cancer is the most frequent malignancy in women worldwide and one of the most curable cancers if diagnosed at an early stage. Female patients presenting solid breast lesions are greatly predisposed to breast cancer development, and as such, effective screening of high-risk patients is valuable in early-stage breast cancer detection. Objectives: The aim of our study was to identify the most relevant demographic, reproductive and lifestyle risk factors for breast cancer among women with solid breast lesions living in western Romania, namely the urban region consisting of Timisoara and the rural surrounding regions. Methods: From January 2017 to December 2021, 1161 patients with solid breast lesions, as detected by sonoelastography, were divided into two groups: patients with benign lesions (1019, 87.77%) and patients with malignant nodules (142, 12.23%). The malignancy group was confirmed by a histopathological result. Variables including age, BMI, menarche, menopause, years of exposure to estrogen, number of births, breastfeeding period, use of oral combined contraceptives, smoker status, family medical history and living area (rural-urban) were recorded. Results: It was evidenced by our study that the main risk factors for malignancy were elevated age (OR = 1.07, 95% CI 1.05–1.08), BMI (OR = 1.06, 95% CI 1.02–1.10), living area (rural) (OR = 1.86, 95% CI 1.13–2.85) and family medical history (negative) (OR 3.13, 95% CI 1.43–8.29). The other proposed risk factors were not found to be statistically significant. Conclusions: Age and BMI were observed to be the most significant factors for breast cancer risk increase, followed by living in a rural area. A family history of breast cancer was shown to be inversely correlated with cancer risk increase.

## 1. Introduction

Breast cancer is a multifactorial disease, one of the most frequent but also one of the most curable if diagnosed in an incipient state [1,2,3]. Various demographic and reproductive factors were shown to be linked to its occurrence [4,5]. Although breast cancer is widespread, there is a variation between its incidence, mortality and survival rates among different countries and regions of the same country. This could be due to multiple factors such as population age distribution, lifestyle, genetic factors, environment and, finally, access to medical services and screening programs [6,7]. According to Globocan 2020, breast cancer is the leading cause of cancer mortality among women in Romania, with a proportion of 26.9% of all cancer-related deaths [8]. Overall, breast cancer cases in Europe are expected to increase to over 560,000 by 2040 from the base value of 531,000 in 2020 [8].

Effective screening and early identification of high-risk patients are the foundation of successful prevention and treatment [9]. The risk classification based on risk factors is a cost-effective method that can improve screening outcomes, resulting in targeted breast cancer screening programs [10]. The identification of benign breast lesions has become more common with the use of sonoelastography as a routine screening technique [11,12]. Women with benign breast lesions already have an increased risk of subsequent breast cancer [13,14,15], and thus, accurate risk estimates are welcomed. As per WHO recommendations, appropriate widespread coverage of high-risk groups is to be preferred compared to repetitive screening of low-risk groups to aid in early cancer detection [16,17].

Previous studies demonstrated that various risk factors including demographic, reproductive, hormonal, hereditary and breast-related factors and lifestyle contribute to breast cancer occurrence [5,6,18,19,20]. On the other hand, protective factors against breast cancer were also identified. These include prolonged breastfeeding (>1 year), higher number of births and late menarche combined with early menopause (shortened period of exposure to estrogens) [3,21]. In the case of other factors, such as smoking status [19,22], the use of combined oral contraceptives and age at first birth, the influence is disputed [4,23,24]. Data are also available on these associations for women explicitly exhibiting benign breast nodules [7,13,25], but a suitable risk classification, adapted to the target populations’ profile, is still desirable. This should aid in improving the selection criteria for further referral and follow-up of patients exhibiting benign breast nodules. The aim of our study is to identify the influence of the independent risk factors (age, BMI, menarche, menopause, years of exposure to estrogen, number of births, breastfeeding period, use of oral combined contraceptives, smoker status, family medical history of breast cancer and rural-urban split) among women with solid breast lesions in our geographical area.

## 2. Materials and Methods

We conducted a retrospective study in our endocrine unit in Timisoara on patient data collected between January 2017 and December 2021. From a total of 3500 female patients examined during the five-year period, we selected 1161 women based on the presence of at least one solid breast lesion as detected by sonoelastography. Of the 1161 patients selected for the study, 1019 patients were subsequently diagnosed with benign breast nodules and 142 were found to exhibit malignant breast nodules following histopathological examination.

The main causes of patient presentation were the presence of a breast lump as detected by auto examination, breast tenderness and a positive medical history of breast cancer. Patients with normal breast and cystic lesions were excluded (BIRADS score 1 and 2). Anamnestic data alongside ultrasound report data were collected for each patient.

The ultrasound imaging technique used (ducto-radial echography) was based on the lobar anatomy of the breasts with the patient lying in a supine position holding both hands above the head. The breast examination was conducted with the nipple positioned in the left upper corner and the peripheral lobar structure in the right part of the screen. All recommended layers were visualized on the screen starting from the upper layers representing the skin followed by the lower layers representing the rib structures and pleura [26]. The equipment used for performing ultrasound investigations was a Hitachi Preirus sonographer (Hitachi Medical Corporation, Tokyo, Japan) equipped with a specific breast probe (EUP-L53L, 920 mm width) employing a water bag device. Doppler and elastography software were included.

Each patient was subjected in the same session to bilateral breast evaluation by means of conventional B-mode ultrasonography, followed by color Doppler scanning and strain elastography. All ultrasound examinations were performed prior to any surgical procedure by an operator with more than 10 years of experience (D.S.) in the field of sonoelastography [27].

Based on the ultrasonography result, a BIRADS (Breast Imaging Reporting And Data System) score was given according to the ACR (American College of Radiology) malignancy criteria [28]. Additionally, resulting from the strain elastography, using both the qualitative and semi-quantitative methods, the color code Tsukuba (ES) and FLR (Fat-to-Lesion Ratio) values were determined. Each solid nodule was measured at least twice in the radial and antiradial direction.

We used the current recommendations given by EFSUMB guidelines where stiffness was considered an extra risk factor [28]. Nodules with BIRADS scores of 4A and below were downgraded if identified as exhibiting low stiffness (FLR < 4 and ES < 4). Any nodule attributed a score of 4B, 4C and 5 was not eligible for downgrade, regardless of the nodule stiffness.

The criteria for referring to biopsy was a BIRADS score of 4B and above. All histopathological examinations were performed by a pathologist specialized in breast tissue diagnosis. Ultrasonography and elastography results of the patients were not available to the pathologist. Malignancy was determined by using the classification as defined by the National Cancer Institute [29].

Patients presenting nodules characterized as BIRADS 4A and 3 were referred to a follow-up after 6 to 12 months following initial investigation.

For the current study, subjects were divided into two groups: patients with benign breast lesions and patients with malignant breast lesions. Malignancy was established by a positive histopathological result.

The study encompasses both continuous and categorical independent variables. Continuous variables comprise age (in years), BMI (in kg/m^2^), menarche onset (in years) and years of estrogen exposure, indicating the fertility duration. Categorical variables encompass living area (urban or rural), smoking status, family history of breast tumors, menopausal status (yes or no), past childbirth, breastfeeding history (yes or no) and past use of combined oral contraceptives (yes or no).

### Statistical Analysis

In order to summarize the characteristics of the study population, we conducted a comprehensive statistical analysis. Continuous variables were expressed as mean ± standard deviation (SD) for normally distributed data and as median with interquartile range (Q25-Q75) for non-normally distributed data. Categorical variables were presented as frequencies and percentages. Normality of continuous variables was assessed using the Shapiro–Wilk test.

Statistical significance between women with benign and malignant tumors was determined using Student’s *t*-test for normally distributed data and the Mann–Whitney U test for non-normally distributed data. The magnitude of the differences between the two groups in terms of continuous variables was assessed using the rank-biserial correlation coefficient. Differences in percentages were assessed using Pearson’s chi-squared test.

To identify independent risk factors for mammary carcinoma, we conducted multivariate logistic regression. Feature selection was performed using the backward elimination method, and model performance was evaluated using the AIC (Akaike Information Criterion) and Nagelkerke’s R^2^. ROC (Receiver Operating Characteristics) curve analysis was used to assess the model performance and to determine the threshold values for the independent risk factors. The cutoff point that optimizes sensitivity and specificity was determined using the Youden index, and the CI (confidence interval) for the AUC (area under the ROC curve) was calculated using the robust DeLong method. Data collection, processing and analysis were carried out with the R software (R Core Team, 2023). Results were presented in tabular and graphical form. Statistical significance was determined using a significance level of *p* < 0.05, with a 95% confidence interval.

## 3. Results

### 3.1. Differences between the Two Groups

The sample comprises 1161 women, all diagnosed with breast tumors. A significant majority of the patients live in urban areas (79.07%), are active smokers (73.64%), have a family medical history (87.60%) and are in menopause (79.76%). Moreover, the majority have never used oral contraceptives (70.28%). More than half of them gave birth before their tumor diagnosis (62.02%), and more than half never breastfed (51.85%).

We observed statistically significant differences between the two groups (benign vs. malignant) regarding their living area (*p* = 0.01), family medical history (*p* < 0.01), menopause (*p* < 0.001), pregnancy status (*p* < 0.001), breastfeeding (*p* < 0.001) and oral contraceptive use (*p* = 0.02). There was no statistically significant evidence indicating a difference regarding the smoker status between the two groups (*p* = 0.47). The results are summarized in Table 1.

There are a wide range of ages among the women in our study, spanning between 13 and 87 years old, with a median age of 41 years. The median BMI was 22.48 kg/m^2^, the median age at menarche was 13 years, and the median duration of exposure to estrogen was 28 years. Using the Shapiro–Wilk test to assess the normality of the distribution, we can observe that none of our numerical variables have followed a Gaussian distribution (*p* < 0.05).

**Figure 1:**
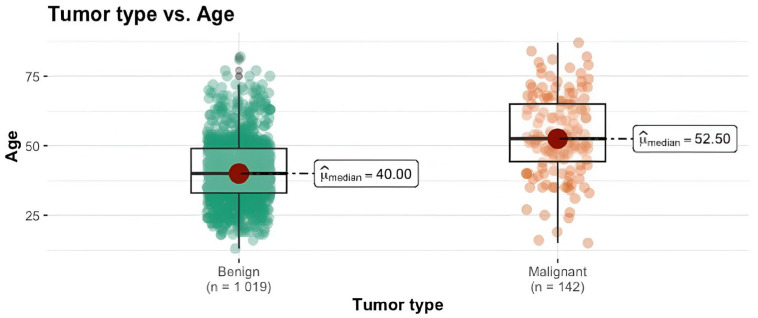
Tumor type vs. age.

**Figure 2:**
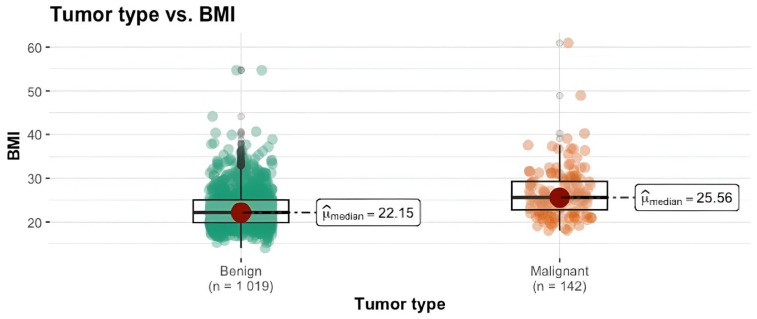
Tumor type vs. BMI.

**Figure 3:**
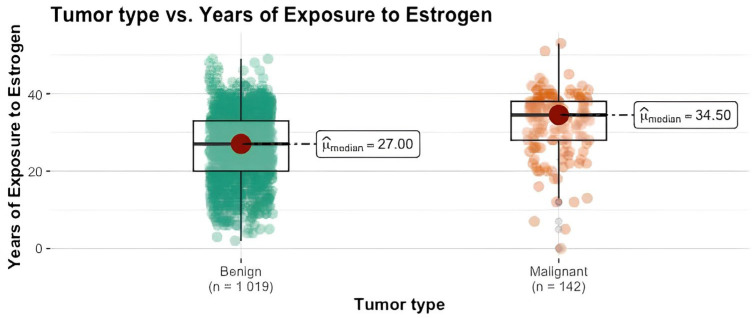
Tumor type vs. years of exposure to estrogen.

We conducted a Mann–Whitney U test to assess the differences between the two groups regarding age, BMI, menarche and years of exposure to estrogen. We can see that the mammary carcinoma group have notably higher median values compared to the benign tumor group, highlighting a statistically significant difference between these two groups (*p* < 0.001). The magnitude of the difference was substantial for age (= −0.50) and BMI (= −0.42), indicating a considerable impact. Regarding years of exposure to estrogen, the effect size was large (= −0.39), while for the menarche, the difference was small (= −0.12). The results are summarized in Table 2 and Figure 1, Figure 2 and Figure 3.

### 3.2. Independent Risk Factors for the Diagnosis of Mammary Carcinoma

In order to identify the independent risk factors that can predict mammary carcinoma, we used multivariate logistic regression. Initially, the model had several independent variables: age, BMI, menarche onset, menopausal status, years of exposure to estrogen, past childbirth, breastfeeding history, past use of oral combined contraceptives, smoker status, family medical history and living area in relation to the dichotomous dependent variable (benign or malignant).

To build the prediction model, we used the backward elimination method for feature selection, Akaike Information Criteria (AIC) to choose the best model and Nagelkerke’s R^2^ to evaluate the performance of the model.

We initiated our analysis by evaluating the multicollinearity among our model predictors, a crucial step to ensure the reliability of our results. To accomplish this, we employed the Variance Inflation Factor (VIF) for each predictor variable. VIF values serve as indicators of multicollinearity: those below 5 suggest insignificant multicollinearity, while values between 5 and 10 indicate a moderate level, and values exceeding 10 raise concerns of severe multicollinearity. The detailed findings of this assessment are summarized in Table 3.

Upon analyzing our results, we observe that age exhibits a VIF of 5.42, signaling moderate multicollinearity. Conversely, all other predictors showcase VIF values below 5, indicating an absence of noteworthy multicollinearity concerns. Consequently, considering these VIF metrics collectively, there is no indication of severe multicollinearity within our regression model.

In the seven-factor model, we included age, BMI, YEE (years of exposure to estrogens), childbirth, family medical history, living area and the use of COC (combined oral contraceptives), and the results reveal a Nagelkerke’s R^2^ of 0.220 (the factors explaining 22% of the variance for the diagnosis of mammary carcinoma) and an AIC of 772.397. The results are shown in Table 4.

We observe that, regarding age and BMI, for each one-unit increase in age, the odds of developing carcinoma increase by approximately 7% and 6%, respectively. In the case of YEE, it does not show a statistically significant association with the outcome (*p*-value = 0.377, *p*-value = 0.855), suggesting a negligible change in the odds for each unit change in YEE. Regarding births, the odds ratio suggests an increase in the odds of developing mammary carcinoma by approximately 33%, but it does not show a statistically significant association with the outcome (*p*-value = 0.249). Our findings also reveal that individuals living in a rural environment setting have 79% higher odds of developing mammary carcinoma compared to urban areas, and individuals with a negative family medical history have 229% higher odds of developing carcinoma compared to those with a positive family history. Regarding the past use of oral contraceptives, the odds ratio suggests an increase in the odds of developing mammary carcinoma by approximately 1%, but it does not show a statistically significant association with the outcome (*p*-value = 0.855).

The optimal model identified is the four-factor model that includes age, BMI, living area and family medical history as independent variables, with a Nagelkerke’s R^2^ of 0.218 (explaining 21.8% of the variation for the diagnosis of mammary carcinoma) and an AIC of 768.287. Despite the fact that the seven-factor model yields superior results in terms of Nagelkerke’s R^2^, the evidence suggests that the four-factor model is superior overall. The results are shown in Table 5 and Table 6.

The risk of developing mammary carcinoma increases with advancing age, higher BMI, residing in a rural environment and among individuals without a family medical history of the condition.

Elevated age is associated with an increased risk of discovering mammary carcinoma by 1.07 times (1 unit increase in age is associated with a 7% increase in the odds of detecting a malignant tumor), signifying a higher risk with advancing age. Similarly, higher BMI scores lead to an increased risk of discovering mammary carcinoma by 1.06 times (1 unit increase in BMI score is associated with a 6% increase in the odds of a tumor being malignant). We also observe that the odds of carcinoma occurrence in the rural environment are 86% higher compared to the reference category (urban). Additionally, patients with a negative family medical history had approximately 213% higher odds of discovering malignant tumors compared to those with a positive family medical history.

### 3.3. Threshold Value for Age and BMI Providing Positive Diagnosis for Mammary Carcinoma

**Figure 4:**
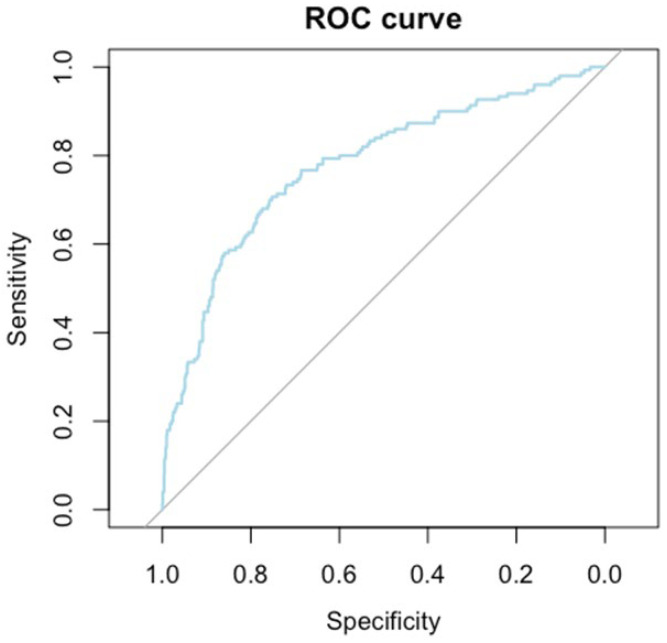
ROC curve for the four-factor model.

AUROC statistics were used to evaluate the performance of our classifier and to determine the threshold values for age and BMI regarding the diagnosis of mammary carcinoma using our four-factor model. The cutoff point (87.219%) was determined using the Youden index (0.459) and the CI for the AUC was determined using the DeLong method. The results of the AUROC analysis are shown in Table 7 and Figure 4.

Those metrics provide a comprehensive evaluation of the diagnostic model, demonstrating good discriminative ability (AUC = 0.766), sensitivity (70.6%), specificity (75.2%) and overall accuracy (74.6%). The threshold values for age and BMI indicate the points at which the model distinguishes between positive and negative cases. The significant *p*-value (*p*-value < 0.001) supports the reliability of the model’s performance.

## 4. Discussion

The findings of previous studies demonstrated that various risk factors including demographic, reproductive, hormonal, hereditary, lifestyle and breast-related factors contribute to the incidence of breast cancer [6]. Additionally, women with benign breast disease have an increased risk of breast cancer development [30] with a relative risk of 1.5 to 1.6 for women with benign breast disease as compared to women in the general population [31]. Although benign breast disease does not necessarily lead to breast cancer, certain risk factors may increase the likelihood [19].

Age is one of the most important known risk factors for breast cancer [32]. Peak breast cancer incidence was observed in menopausal women with breast cancer risk being directly correlated with patients’ elevated age [6,33,34,35]. Our results are in line with the existing literature, showing that elevated age is a risk factor for breast cancer (each year adding a further 7% increase in the risk of malignancy). The median age for the cancer group is 52.5 compared to 40 for the benign group, further confirming that breast cancer risk increases with age and is found mostly in menopausal women.

BMI is another well-established predictor of breast cancer development [32]. Previous studies have shown elevated BMI to be a risk factor for breast cancer development [36,37,38,39]. We found a similar correlation between breast cancer incidence and elevated BMI (each unit increase in BMI leads to an increase in the odds of cancer by 7%). Median BMI in the cancer group was 25.56 compared to 22.15 for the benign group. The BMI threshold value of 28.5 strengthens the hypothesis that overweight women are clearly at risk of developing breast cancer.

In general, the literature describes urban living as a risk factor, increasing the likelihood of breast cancer [40]. Our study, on the other hand, shows that patients living in a rural environment are more likely to be diagnosed with breast cancer. One hypothesis for why this is the case is that rural patients, on average, are more likely to be diagnosed at a late stage compared to urban residents [41,42,43]. It is important to note, however, that women in rural areas have poorer access to healthcare and on average are less educated, thus furthering late-stage presentation [44,45,46]. The results of a recent review article on breast cancer risk indicate that social determinants, such as poverty, lack of education, lack of social support and social isolation, play an important role in the stage at diagnosis and decreased survival [44].

Overall, breast cancer patients in the majority of low- and middle-income countries were found to be diagnosed with a later stage of breast cancer compared to the ones living in high-income countries [47]. On top of this, breast cancer screening program availability and coverage varies from country to country. A recent EU-wide study has placed the total examination coverage between 49% in the Eastern EU area and 69% in the Southern EU area [48]. As our study was conducted in Romania, a country where the average income closely correlates to the urban-rural residence (the urban population on average is wealthier), our results further reinforce why rural patients have a later stage of presentation. Additionally, the geographical location of our country puts our patients in the group with the lowest breast cancer screening availability and coverage.

Another potential cause for the later-stage breast cancer diagnostic for rural patients could stem from psychological factors, namely anxiety from participation in routine breast cancer screening programs. It was shown that the adverse psychological effects of cancer screening are accentuated in individuals belonging to lower-income, skill and education groups [49,50]. As the rural population in the country where our study was conducted is on average less educated, later addressability might also be influenced by increased negative psychological factors affecting rural patients.

The differences in exposure to environmental factors between rural and urban populations should not be omitted. One particular example is that of exposure to endocrine disruptors, as found in some agrochemical pesticides, in the case of rural residents. Several studies showed potential links between breast lesion formation and exposure to such chemicals [51,52,53]. Unfortunately, we do not have any clear confirmation of patient exposure to agrochemical pesticides and can only assume that, in the case of rural residing patients, the likelihood of such exposure is higher than for urban residents. Finally, we do not believe the higher likelihood of cancer for rural patients in our study to be linked to the environment per se, but rather it is a consequence of the socio-economic profile of the rural inhabitant characteristic of our geographical location. This hypothesis fits with previously published results [54], which showed that socio-economic conditions will ultimately influence the likelihood of breast cancer, specifically breast cancer diagnosed at a late stage, independently of rural-urban living environment. Better screening for breast cancer is required in closing the gap between rural and urban patient outcome and early breast cancer detection [48,55].

Family medical history was also found to be a relevant risk factor for breast cancer [6]. In this case, however, our results show a reverse correlation to what is described in the existing literature [56,57]. The consensus is that patients with a positive family history of breast cancer are at risk of developing breast cancer [58]. As described in the results, we identified a positive increase in breast cancer incidence associated with patients who have negative family medical history. We must highlight that the positive family medical history for breast cancer among first- and second-degree relatives is based only on patients’ anamnestic data without confirmation by histopathological results.

One of the hypotheses to explain this mismatch is that patients with a positive family history of breast cancer are more likely to participate in regular screening programs as evidenced in previous studies [57,59,60,61]. Our study shows that 87.60% of patients included in the study declare that they have at least one first- or second-degree relative with breast cancer. This could be explained by the fact that women with a known family history of breast cancer are more likely to take a proactive stance and refer to medical assistance as soon as any suspicious symptoms appear.

Another hypothesis is that although the breast cancer risk is still elevated for patients with a positive family history of breast cancer, the age at presentation for the population in our study is lower than the high-risk age group (median age for benign patients is 40 compared to 52.5 for cancer). In this case, we consider that patients with a positive family history of breast cancer and exhibiting benign nodules are still considered at risk for malignancy and included in screening programs [16,62,63,64].

The other proposed demographic, reproductive and lifestyle factors, as well as age, BMI, living area and family medical history of breast cancer, were not found to be statistically significant by our study. However, they should not be discarded as multiple studies have shown them to be linked to an increased risk of breast cancer [6,20]. Early menarche and late menopause (prolonged exposure to estrogen) were shown to be significant risk factors for breast cancer development [21,32,65]. On the other hand, early full-term pregnancy and prolonged breastfeeding were shown to be protective factors, decreasing the risk of breast cancer development [21,66]. Both in our results and in other existing published literature, smoking was not shown to be of statistical relevance regarding an increase in breast cancer risk [19,22]. Similarly, for the use of combined oral contraceptives, we identified no link to breast cancer risk increase. Some studies have highlighted a connection between breast cancer incidence and length of combined oral contraceptive use [23], whereas in others, this link was not present [24].

Overall, in our study, age and BMI were found to be most strongly associated with an increased risk of breast cancer, followed by living area and a family history of breast cancer. Patients with benign breast nodules who exhibit these risk factors should be followed up with rigorously.

The contribution of our study was to show a feature that is characteristic for our region, western Romania, namely that menopausal, overweight patients coming from rural areas and exhibiting solid breast lesions are at a greater risk of being diagnosed with breast cancer. This finding reflects a lower level of patient addressability among women living in rural areas combined with a potentially unhealthier lifestyle as indicated by the increased BMI values. Despite a lower number of the overall cohort, patients coming from rural areas are overrepresented in the malignant group (OR 1.86, CI 1.19–2.85, *p* = 0.005), indicating the need to consider this factor when proposing risk classification systems.

Some weak points of the study are that not all of the patients that were referred for follow-ups respected this recommendation and a potential later diagnostic upgrade requiring referral to biopsy was missed. Socioeconomic factors related to discrepancies between rural and urban residents were assumed based on the general population and were not collected for each patient. The history of patients’ exposure to chemicals, such as in the case of agrochemical pesticides containing endocrine disruptors, was not collected.

Further research directions should be pointed at developing screening and follow-up programs adapted to the local profile of the population, considering the characteristic risk profile.

## Figures and Tables

**Table 1:** Categorical variables.

Variable	Yes	Proportion	No	Proportion	Chi-Squared
Living area [Urban]	918 (79%)	Bn 652 (71%)	243 (21%)	Bn 224 (92%)	0.01
		Mg 266 (29%)		Mg 19 (8%)	
Smoker status	306 (26%)	Bn 263 (86%)	855 (74%)	Bn 752 (88%)	0.47
		Mg 43 (14%)		Mg 103 (12%)	
Family medical history	144 (12%)	Bn 138 (96%)	1017 (88%)	Bn 875 (86%)	<0.01
		Mg 6 (4%)		Mg 142 (14%)	
Menopause	235 (20%)	Bn 167 (71%)	926 (80%)	Bn 852 (92%)	<0.001
		Mg 68 (29%)		Mg 74 (8%)	
Gave birth	720 (62%)	Bn 605 (84%)	441 (38%)	Bn 415 (94%)	<0.001
		Mg 115 (16%)		Mg 26 (6%)	
Breastfeeding	559 (48%)	Bn 470 (84%)	602 (52%)	Bn 554 (92%)	<0.001
		Mg 89 (16%)		Mg 48 (8%)	
Oral contraceptive use	345 (30%)	Bn 311 (90%)	816 (70%)	Bn 702 (86%)	0.02
		Mg 34 (10%)		Mg 114 (14%)	

Observations—1161; benign tumor—1019 (87.77%); malignant tumor—142 (12.23%); chi-squared—*p*-value for chi-squared test; Bn—benign tumor; Mg—malignant tumor.

**Table 2:** Continuous variables.

Variable	Median	Q25–Q75	Shapiro–Wilk	Mann–Whitney U	r
Age	41.00	34–51	<0.001	<0.001	−0.50
BMI	22.48	20.02–25.60	<0.001	<0.001	−0.42
Menarche	13.00	12.00–14.00	<0.001	0.01	−0.12
Years of exposure to estrogen	28.00	20.00–34.00	<0.001	<0.001	−0.39

Abbreviations: BMI—Body Mass Index; Q25–Q75—interquartile range; Shapiro–Wilk—*p*-value for Shapiro–Wilk test; Mann–Whitney U—*p*-value for Mann–Whitney U test; r—rank-biserial correlation coefficient.

**Table 3:** Multicollinearity assessment.

Variable	VIF
Age	5.42
BMI	1.18
Menarche	1.20
Menopause	2.98
Years of exposure to estrogen	2.60
Gave birth	2.43
Breastfeeding	2.65
Oral contraceptive use	1.84
Smoker status	1.05
Family medical history	1.01
Living area	1.03

Abbreviations: BMI—Body Mass Index.

**Table 4:** Logistic regression for determining independent risk factors in the seven-factor model.

Predictors	Odds Ratio	95% CI	*p*-Value
Age	1.07	1.05–1.09	<0.001
BMI	1.06	1.02–1.10	0.001
YEE	0.99	0.95–1.02	0.377
Childbirth [Yes]	1.33	0.83–2.18	0.249
Living area [Rural]	1.79	1.16–2.73	0.007
FMH [Negative]	3.29	1.50–8.71	0.007
COC [Yes]	1.01	0.94–1.07	0.855

Abbreviations: BMI—Body Mass Index; FMH—family medical history; YEE—years of exposure to estrogens; COC—past use of combined oral contraceptives; CI–95% confidence interval.

**Table 5:** Comparison between the seven-factor model and four-factor model.

Model	Nagelkerke’s R^2^	AIC
Seven-factor model	0.220	768.287
Four-factor model	0.218	772.397

**Table 6:** Logistic regression for determining independent risk factors on four-factor model.

Predictors	Odds Ratio	95% CI	*p*-Value
Age	1.07	1.05–1.08	<0.001
BMI	1.06	1.02–1.10	0.001
Living area [Rural]	1.86	1.19–2.85	0.005
FMH [Negative]	3.13	1.43–8.29	0.010

Abbreviations: BMI—Body Mass Index; FMH—family medical history; CI—95% confidence interval.

**Table 7:** AUROC analysis of the four-factor model.

AUC (CI—DeLong)	0.776 (0.733–0.819)
Sensitivity	70.6%
Specificity	75.2%
Accuracy	74.6%
*p*-value	<0.001
Age threshold value	58
BMI threshold value	28.53

Abbreviations: AUC—area under the ROC curve; BMI—Body Mass Index; CI—confidence interval.

## Data Availability

The data that support the findings of this study are available on reasonable request from the corresponding authors. The data are not publicly available due to privacy or ethical restrictions.
